# Metabolic and Proteomic Reveals of 7Li (Lithium-7) Ion Beam Radiation in *Capsicum annuum* L.

**DOI:** 10.3390/genes16121486

**Published:** 2025-12-12

**Authors:** Yue Huang, Maojingkai Li, Yan Li, Xingliang Wang, Chongyu Gu, Jianzhong Wu, Xue Wang

**Affiliations:** 1 Horticultural Branch, Heilongjiang Academy of Agricultural Sciences, Harbin 150069, China; 2 College of Horticulture, Northeast Agricultural University, Harbin 150030, China; 3 Grassland Research Institute, Heilongjiang Academy of Agricultural Sciences, Harbin 150086, China

**Keywords:** *Capsicum annuum* L., 7Li ion beam radiation, metabolic, proteomic

## Abstract

Background: Chili pepper (*Capsicum annuum* L.), a globally cultivated and ancient domesticated crop, carries considerable significance in agriculture. While radiation-induced mutagenesis has found application in this crop, the mutagenic efficacy and molecular-level impacts of 7Li ion beam radiation remain poorly elucidated. Methods: We irradiated pepper with a beam of 7Li ions to create a mutant, which showed good economic traits, and phenotypic and physio-biochemical characterization were combined with proteomic and metabolomic profiling to delineate the mutagenic mechanisms. Quantitative real-time PCR (qRT-PCR) was further utilized to assess the biological impact and underlying response pathways. We used this to evaluate the biological impact and the reaction mechanisms behind it. Results: 7Li beam radiation positively influenced morphology and physiological traits, notably chlorophyll and anthocyanin content. Leveraging proteomic profiling detected 6082 proteins, including 355 differential proteins (139 upregulated, 216 downregulated), enriched in 4 KEGG pathways. Based on GO and KEGG network analysis, 250 metabolites were quantified, with 120 being differentially abundant (112 upregulated, 8 downregulated), enriched in 9 metabolic pathways. Furthermore, qRT-PCR results revealed that differentially expressed genes were consistent with the corresponding metabolomic data. Joint analysis revealed the coordinated enrichment of differential metabolites and proteins in pathways related to amino acid and carbohydrate metabolism. These findings suggest that these active pathways in pepper are related to its response to ion beam radiation. Overall, this study is a valuable resource for subsequent genomic research on peppers and 7Li ion beam radiation research.

## 1. Introduction

Chill pepper is a globally cultivated vegetable crop of significant agricultural and economic importance, serving dual purposes as a fresh vegetable and processed food ingredient, while also functioning as an essential spice [1]. In recent years, the cultivation area in China for pepper has steadily expanded, accounting for approximately 8–10% of China’s total vegetable planting area. The industry exhibits increasing regional specialization, with a growing diversity of cultivation systems and consumer-oriented varieties, alongside a notable rise in the production of high-quality pepper types [2]. Consequently, breeding new pepper cultivars with high yield, superior quality, multi-stress resistance, and broad adaptability is important to critical agriculture systems.

Nowadays, people find many ways to create new germplasms, and mutation breeding is a tool for novel crop cultivar development. In recent years, ion beam radiation has become one of the most widely used mutagenic breeding techniques [3,4]. Xiong et al. [5] utilized 7Li ion beam radiation generated by the HI-13 tandem accelerator to directly irradiate crop seeds. Their study revealed that nuclear reactions of 7Li ion beams within the seed embryos produced 7Be nuclides, causing irreparable damage to hydrogen bonds in DNA molecules. Compared with other mutagenesis techniques, 7Li ion beam radiation induces a higher frequency of single-base substitutions, thereby generating diverse mutant phenotypes [6]. This process increased mutation rates, thereby generating novel germplasm for breeding applications. 7Li ion beam radiation has been shown to have high agronomic relevance for cultivar development, and this status primarily stems from its operational advantages: it maintains a high rate in generating diverse mutations, while concurrently minimizing physiological injury to plant tissues and ensuring the stable inheritance of the induced genetic alterations [7,8,9].

7Li ion beam radiation is now widely used in mutation breeding research, with successful implementations reported in diverse species including Arabidopsis, maize, and wheat [10]. Wang et al. [11] radiated rice seeds with 7Li ion beams and γ-rays. They observed that in the M2 generation, 7Li ion beam radiation induced a higher frequency of chlorophyll-deficient, plant height, and heading date mutations, with a greater proportion of beneficial trait mutations compared to γ-ray treatment. In summary, scientists compared various ion beam species and doses—delivered to plants, seeds, and other materials—with conventional radiation have demonstrated that 7Li ion beam radiation offers distinct advantages, including reduced physiological damage, and high mutation frequencies [7,12,13]. 

The application of 7Li ion beam radiation has thus played a significant role in crop genetic improvement. However, as a relatively novel mutagenic breeding technique, its underlying mechanisms remain incompletely understood, and its use in pepper breeding has thus far been limited [14,15].

Up to now, there has not been any integrated research that has clearly outlined the concurrent physiology, proteomic, and metabolomics alterations in chili pepper following mutagenic treatment. We therefore compared phenotypic and physiological traits in pepper following 7Li ion beam radiation and employed integrated proteomic and metabolomic analyses to elucidate the underlying molecular mechanisms. These findings enhance our understanding of 7Li-induced molecular variations in pepper and establish a new framework for mutagenesis breeding and germplasm innovation.

## 2. Materials and Methods

### 2.1. Plant Materials

This study utilized two *Capsicum annuum* varieties: the wild-type Long 5 and its mutant derivative Long 158 (generated by 42.3 MeV ion beam radiation at 60 Gy, 6 Gy/min). Our team investigated mutant materials derived from pepper seeds treated with 0–100Gy of 7Li ion beams, identifying the mutant material Long 158 with superior comprehensive agronomic traits [16]. Both genotypes were cultivated at the Horticultural Sub-Academy of the Heilongjiang Academy of Agricultural Sciences (Harbin, China).

### 2.2. Phenotypic Identification and Analysis

A comparative analysis of plant coloration was conducted between the two materials, utilizing three randomly chosen plants from each variety, following initial photographic recording. And conducted transmission electron microscopy observations, as described by Dwiranti et al. [17]. Stem tissue sections (3 mm × 1 mm) of pepper were fixed in 2.5% glutaraldehyde, washed with 0.1 mmol/L phosphate buffer (pH 6.8) three times (15 min each). Post-fixation was performed with 1% osmium tetroxide for 2.5 h, after which samples were washed three times (15 min each) in phosphate buffer to remove residual osmium. Dehydration was carried out with different concentrations of ethanol solutions (50%, 70%, 90%, and 100%) for 15 min per step. Samples were then incubated in a 1:1 mixture of 100% ethanol and 100% acetone for 10 min, followed by pure acetone for 5 min. Infiltration was performed using graded mixtures of acetone and embedding medium (1:1, 1:2, and 1:3), followed by embedding, polymerization, and trimming. Ultrathin sections were stained with uranyl acetate and lead citrate prior to observation and imaging.

Changes in chlorophyll anthocyanidin content in stem tissues were measured according to the method of Inseekp et al. [18]. The specific operation of chlorophyll is as follows: pepper stem tissue (0.3 g) was homogenized with 80% acetone, adjusted to 4 mL with 80% acetone, and extracted in darkness. Following centrifugation at 12,000 rpm and 4 °C for 20 min, the supernatant was collected and brought to a final volume of 10 mL with 80% acetone. Absorbance was measured at 470 nm, 645 nm, and 663 nm using 80% acetone as the blank. Each sample treatment was repeated three times. Calculation formula: Chlorophyll a C_a_ (mg L^−1^) = 12.72A_663_ − 2.59A_645_; Chlorophyll b: C_p_ (mg L^−1^) = 22.88A_645_ − 4.67A_663_; Total chlorophyll: C_t_ (mg L^−1^) = 20.29A_645_ + 8.05A_663_; Carotenoids: C_c_ (mg L^−1^) = (1000A_470_ − 3.27C_a_ − 104C_p_)/229; Pigment content (mg g^−1^ FW) = C × V × N/W; where C is pigment concentration (mg L^−1^), V is total extract volume (L), N is dilution factor, and W is fresh weight (g).

The anthocyanin extraction is as follows: Pepper stem tissue (0.6 g) was flash-frozen in liquid nitrogen, ground to a fine powder, and extracted with 10 mL of methanol–acetic acid (99:1, *v*/*v*) solution for 10 min. After centrifugation at 12,000 rpm for 10 min, 1 mL aliquots of the supernatant were mixed separately with 4 mL of KCl buffer (pH 1.0) and 4 mL of NaAc buffer (pH 4.5). Following 15 min of equilibration, absorbance was measured at 530 nm and 700 nm. Each sample process was repeated three times. Calculation formula: Anthocyanin content (mg L^−1^ FW) = (∆A × MW × DF × 1000)/(W × Ɛ), where ∆A = [(A_530_ − A_700_) at pH 1.0] − [(A_530_ − A_700_) at pH 4.5]; MW = 449.2 g mol^−1^ (molecular weight of cyanidin-3-glucoside); DF = dilution factor; Ɛ = 29,600 L mol^−1^ cm^−1^ (molar extinction coefficient of cyanidin-3-glucoside); and W = fresh weight (g).

This study elucidates the effects of 7Li ion beam radiation on pepper stem tissues.

### 2.3. Identification of Different Resistance Levels of Pepper Seedlings

Uniform seedlings of pepper cultivars Long 5 and Long 158 were transplanted into nutrient pots and maintained under standard cultivation conditions. Plants were grown at ambient temperature (25–35 °C) under natural sunlight, with irrigation every 4 days to maintain soil moisture content at 75–85%. Stress treatments were initiated when plants developed 5 true leaves.

Four abiotic stress treatments were applied as follows.

High temperature stress: Plants were subjected to 40 °C in an illuminated growth chamber for 48 h to simulate extreme heat conditions. Drought stress: After thorough pre-watering, plants underwent complete water withdrawal for 6 days. Cold stress: Detached leaf assays were performed with temperature treatments at 4 °C and −20 °C for 48 h. Salt stress: Plants were treated with 150 mmol/L NaCl solution through root irrigation every 4 days for a total of 5 applications. We conducted photographic documentation of control and treatment groups. Plant wilting severity and leaf rolling degree following stress exposure were quantified (The evaluation criteria for grading plant tolerance to high temperature, drought, low temperature, and salt stress are provided in Supplementary Table S1). Relative water content, photosynthetic fluorescence parameters [19], and the contents of total chlorophyll [18], carotenoids [18], and anthocyanins were measured to assess stress tolerance between Long 5 and Long 158. Three biological replicates were included per sample group. The figure was created using GraphPad Prism 10.6.1, and significance levels were determined by IBM SPSS Statistics 19, with a significance level of 0.05.

### 2.4. Proteomics and Data Analysis

Fresh stem samples (60 mg) were collected for proteomics, which involved three biological replicates for each treatment.

Quantitative proteomics utilizing iTRAQ labeling was performed commercially. Following peptide labeling with an 8-plex kit, samples were pooled, lyophilized, and reconstituted in 0.1% formic acid for LC-MS/MS analysis (Orbitrap Fusion™ coupled to EASY-nLC 1200, Waltham, MA, USA). MS/MS data processing through PEAKS Studio identified differentially expressed proteins using thresholds of |fold change| > 1.5, ≥ 2 unique peptides, and significance score > 20 (*p* < 0.01). Enrichment analyses included GO term assessment via Fisher’s exact test, KEGG pathway analysis using KOBAS, and GSEA implementation following established methodology. The reference genome and protein were obtained from: Pepper Genomics Database. Sequence alignment against this designated reference yielded mapping rates between 95% and 100%.

### 2.5. Untargeted Metabolomics and Data Analysis

Fresh leaf samples (60 mg) were collected for metabolite analysis with six biological replicates per treatment. Untargeted metabolomics analysis was conducted by Shanghai Boyun Biotechnology Co., Ltd. (Shanghai, China) using an Agilent 7890A/5975C GC-MS system. Metabolite extracts were analyzed by liquid chromatography-mass spectrometry (LC-MS). Principal component analysis (PCA) and partial least squares discriminant analysis (PLSDA) were employed to identify differential metabolites across groups. Metabolites were annotated by the KEGG database for functions and pathways analysis. Those with FC > 1.5 or < 0.667 and a *p*-value < 0.05 were considered differentially expressed metabolites (DEMs).

### 2.6. Quantitative Real-Time PCR

Total RNA was extracted from pepper stems using the RNAprep Pure Plant Kit (TianGen, Beijing, China). Reverse transcription into complementary DNA was subsequently performed with the KOD One™ PCR Master Mix reverse transcription kit (TOYOBO, Shanghai, China). Quantitative real-time PCR (qRT-PCR) was conducted using an ABI QuantStudio 3 system (Thermo, Waltham, MA, USA) and 96-well reaction plates under the following parameters: initial denaturation at 95 °C for 3 min, 40 cycles of denaturation at 95 °C for 10 s, and annealing/extension at 68 °C for 15 s. Relative expression of target genes was calculated according to the 2^−ΔΔCT^ method, and primer details are listed in Supplemental Table S2.

## 3. Results

### 3.1. Plant Growth Phenotypes Under Distinct Stress Regimes

**Figure 1:**
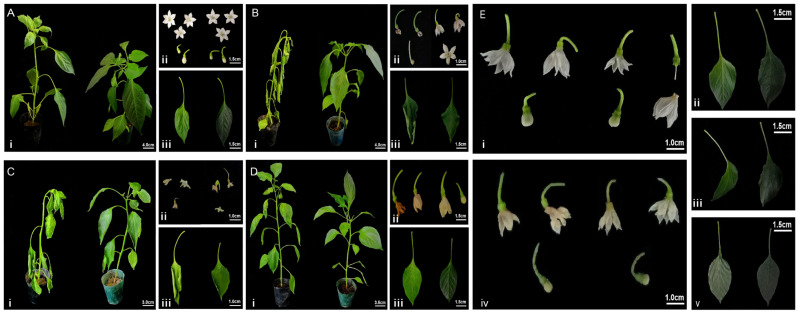
Growth performance of Long 5 and Long 158 cultivars subjected to diverse stress conditions. (**A**) Plants (**i**), flowers (**ii**) and leaves (**iii**) of Long 5 (Left) and Long 158 (Right) under Control. (**B**) Plants (**i**), flowers (ii) and leaves (**iii**) of Long 5 (Left) and Long 158 (Right) under high temperature stress. (**C**) Plants (**i**), flowers (**ii**) and leaves (**iii**) of Long 5 (Left) and Long 158 (Right) under drought stress. (**D**) Plants (**i**), flowers (**ii**) and leaves (**iii**) of Long 5 (Left) and Long 158 (Right) under 150 mM salt stress. (**E**) The flower shape at 4 °C (**i**). Front of the leaf 4 °C (**ii**). The side of the leaf at 4 °C (**iii**). The flower shape at −20 °C (**iv**). The leaf at −20 °C (**v**).

Long 5 exhibited a plant height of 32 cm and a leaf length of 5.25 cm. Following 7Li ion beam irradiation, Long158 showed a reduced plant height of 24 cm but an increased leaf length of 6 cm. Under standard growth conditions, Long 158 exhibited a compact architecture with darker, broader leaves and shorter petioles compared to Long 5, resulting in overall denser foliage (Figure 1A). Floral morphology differed markedly between cultivars: Long 5 produced predominantly hexamerous flowers (rarely pentamerous) with short pedicels, large buds, and smaller corollas, whereas Long 158 formed mainly pentamerous flowers (occasionally hexamerous) with elongated pedicels, smaller buds, and larger corollas.

Analysis of Supplementary Table S1 indicates that Long 5 exhibited a heat damage index of grade 3, whereas Long 158 showed a heat damage index of grade 0. Long 158 demonstrated overall greater thermotolerance. Following 48 h exposure to 40 °C, cultivars exhibited starkly divergent stress responses (Figure 1B). Long 5 plants displayed severe thermodamage, including complete wilting, petiole collapse, apical bending, and chlorotic leaf wrinkling. Floral abortion occurred with petal desiccation and pedicel abscission in fruit-bearing nodes. In contrast, Long 158 maintained turgor pressure despite mild epinasty, exhibiting only marginal leaf curling and transient chlorophyll degradation. While floral retention was observed, few flowers showed pedicel curvature and premature abscission.

Analysis of Supplementary Table S1 shows that Long 5 exhibited a drought damage index of grade 5, while Long 158 had a drought damage index of grade 3, demonstrating that Long 158 is more drought-tolerant than Long 5. After 6d drought stress, cultivars exhibited distinct morphological responses (Figure 1C). Long 5 showed severe wilting with petiole and apical collapse, accompanied by leaf curling, pronounced chlorosis, and necrotic leaf tips. Reproductive development was arrested, evidenced by pedicel shrinkage and complete floral abortion. In contrast, Long 158 maintained partial turgor pressure, displaying only marginal petiole epinasty and leaf margin curling. While floral abscission occurred, few flowers retained viability despite petal wilting and pedicel curvature.

Analysis of Supplementary Table S1 shows that Long 5 had a salt-damage index of grade 3, whereas Long 158 showed a salt-damage index of grade 0, demonstrating that Long 158 exhibits salt-stress tolerance. After 5d NaCl exposure (150 mM), Long 5 exhibited severe salt stress symptoms, including leaf wilting, apical necrosis, and pronounced chlorosis (Figure 1D). Reproductive structures showed developmental arrest, with floral yellowing, premature abscission, and delayed growth during late flowering stages. In contrast, Long 158 maintained near-normal growth architecture, displaying only marginal chlorophyll degradation.

Analysis of Supplementary Table S1 indicates that Long 5 and Long 158 exhibited differential cold-damage responses. Long 5 had a cold-damage index of grade 4, while Long 158 showed a cold-damage index of grade 2. Following 48 h of low-temperature treatments, *Capsicum cultivars* exhibited distinct phenotypic responses (Figure 1E). Under 4 °C exposure, Long 5 displayed leaf epinasty and chlorosis, accompanied by floral closure, developmental delay, and premature abscission. In contrast, Long 158 maintained darker green foliage with only marginal leaf tip curling, though similar floral abscission was observed. At −20 °C, Long 5 developed severe freezing damage with leaf softening and necrosis, coupled with complete floral collapse (petal wilting, pedicel softening, and corolla abscission). Long 158 showed partial tolerance, exhibiting leaf softening without necrosis, though floral development was arrested with visible petal dehydration.

Phenotypic screening under multi-stress conditions revealed that the 7Li ion beam radiation-induced mutant Long 158 consistently exhibited enhanced stress resilience compared to wild-type Long 5 (Figure 1). Systematic evaluation across four abiotic stressors (heat, drought, cold, and salinity) demonstrated superior tolerance in the mutant line.

### 3.2. TEM Analyses of Pepper Stem Cross-Sections

**Figure 2:**
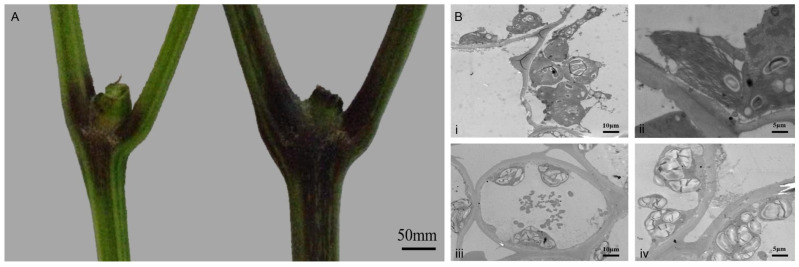
(**A**): stem color between Long 5 (left) and 158 (right). (**B**): Ultrastructure of pepper stem tissues; (**i**): Long158 cell, scale: ×1.5 k, 10.0 μm; (**ii**): Long158 chloroplast, scale: ×2.5 k, 5.0 μm; (**iii**): No. 5 cell, scale: ×1.5 k, 10.0 μm; (**iv**): No. 5 chloroplast, scale: ×2.5 k, 5.0 μm.

Comparative analysis of Long 5 and Long 158 revealed distinct pigmentation differences at the stem nodes (Figure 2A), with Long 158 exhibiting darker stem coloration. Transmission electron microscopy (TEM) of stem tissues showed that chloroplasts in Long 158 cells were predominantly clustered, swollen, and exhibited blurred chloroplast membranes. Thylakoid stacking appeared disorganized, with partial chloroplast fibrosis. In contrast, Long 5 displayed normally shaped and uniformly distributed chloroplasts, with tightly stacked thylakoid membranes.

### 3.3. Contents of Physiological Assays and Enzyme Activity Determination

**Figure 3:**
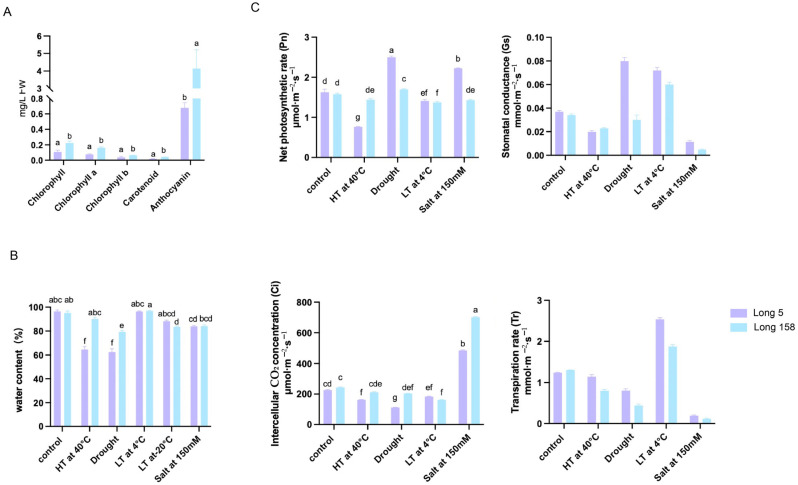
Contents of physiological assays and enzyme activity determination. (**A**) Effect of 7Li ion radiation on pigment content in stem tissue of pepper. (**B**) Changes in relative water content of leaves under different stresses. (**C**) The changes in Pn, Gs, Ci and Tr in leaves under different stresses. Note: Control: no stress; HT: high temperature stress; Drought: drought stress; LT: low temperature stress; Salt: salt stress; Coercion: treatment method. The data represent means for three individual measurements ± SD. Homogeneous groups according to Tukey′s HSD test (*p* < 0.05) are denoted by the same letters.

As shown in Figure 3A, 7Li ion beam radiation significantly altered pigment profiles in pepper stem tissues. Total chlorophyll, chlorophyll a, chlorophyll b, carotenoid, and anthocyanin contents were markedly higher in Long 158 than in Long 5 following treatment.

Following heat and drought stress (Figure 3B), Long 5 exhibited a significant reduction in leaf water content, whereas Long 158 maintained relatively stable levels under these conditions, indicating stronger stress tolerance in the latter. We measured photosynthetic performance and chlorophyll fluorescence parameters in both Long 5 and Long 158 under various stress conditions, while also assaying the activity levels of key stress-responsive enzymes (Figure 3C). Under control conditions, the two varieties exhibited comparable chlorophyll fluorescence parameters and key photosynthetic traits, including net photosynthetic rate, transpiration rate, intercellular CO_2_ concentration, and stomatal conductance. Drought stress induced a slight decline in both cultivars. Following heat stress, Long 5 exhibited increased maximum net photosynthetic rate (Pnmax) and stomatal conductance (Gs), but reduced intercellular CO_2_ concentration (Ci) and transpiration rate (Tr), whereas Long 158 showed no significant alterations. Low-temperature treatment significantly elevated Gs and Tr in both cultivars. After 150 mM NaCl treatment, Long 5 displayed a marked increase in Pnmax, but a significant decrease in Gs and Tr, while Long 158 exhibited no notable changes in photosynthetic parameters except for significantly reduced Gs and Tr.

### 3.4. GO Enrichment and KEGG Pathway Analyses of DEPs Response to Long 5 and Long 158

**Figure 4:**
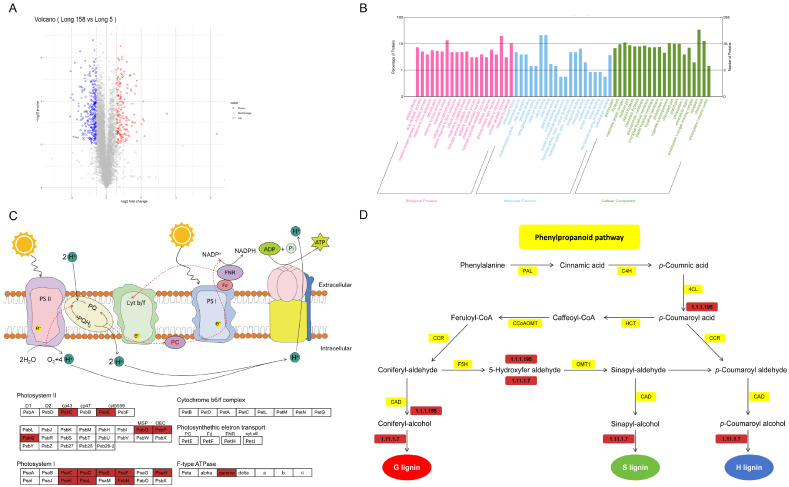
Map of proteomic profiles. (**A**) Volcanic distribution map of up-regulated and down-regulated proteins. (**B**) GO annotation of differentially expressed proteins. (**C**) KEGG pathway map of up regulated proteins (red) in photosynthesis. Red indicates up regulated. (**D**) KEGG pathway map of up regulated protein (red) in Phenylpropane biosynthesis. Red indicates up regulated.

GO enrichment analysis was also conducted on differential proteins (DEPs) between Long 5 and Long 158, and the identification of DEPs between Long 5 and Long 158. A total of 60 GO terms were enriched in three ontologies, namely, cellular components, biological processes, and molecular function. A total of 20 classes in the biological process were enriched in terms of response to stimulus (19.44%), response to stress (13.52%), and response to chemical (10.42%). A total of 20 classes were found for the molecular function, which include metal ion binding (21.13%), cation binding (21.13%) and tetrapyrrole binding (6.48%). A total of 20 classes existed in the cellular component category, which include in cytoplasm (30.80%), chloroplast (12.68%) and membrane protein complex (10.99%). (Figure 4B)

Among the 72 KEGG pathways identified for the 271 DEPs, the biosynthesis of photosynthesis-related genes displayed the highest abundance. Concurrently, a significant presence within phenylpropanoid biosynthesis suggests that these proteins are principally associated with this metabolic process in pepper stems. That means that 7Li ion beam radiation can promote pepper growth. In relation to this, photosystem I (PS I) and photosystem II (PS II) were analyzed by KEGG pathway clustering to further determine the photosynthetic responses of 7Li ion beam radiation. In both PS I and PS II, all Long 158 DEPs were significantly upregulated (Figure 4C). Proteomic profile analysis revealed coordinated upregulation of phenylpropanoid biosynthesis enzymes, including cinnamyl-alcohol dehydrogenase (CAD) and class III peroxidases ([EC:1.11.1.7]) (fold change 1.69, *p* value 0.0000794) (Figure 4C).

### 3.5. Differentially Accumulated Metabolites in Capsicum annuum L.

**Figure 5:**
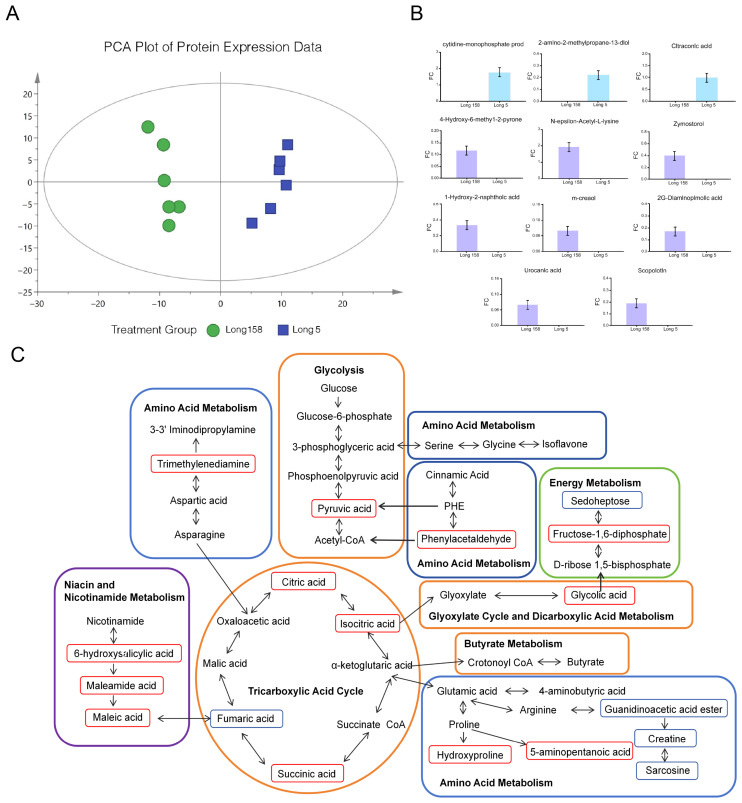
Metabolites between Long 5 and Long 158. (**A**) PCA scores of samples. (**B**) Differential metabolites between Long 5 and Long 158. (**C**) Metabolic pathway map of differential metabolites. Note: In the figure, the orange area is glucose metabolism, the blue area is amino acid metabolism, the green area is energy metabolism, the purple area is niacin and niacinamide metabolism, and the red border is marked with up-regulated expression compounds.

Non-targeted metabolomics identified 120 major different metabolites (Supplementary Table S2). PCA revealed a robust separation between Long 5 and Long 158, which was further confirmed by a distinct OPLS-DA (Orthogonal Partial Least Squares Discriminant Analysis) clustering, validating the model’s reliability in assessing inter-cultivar metabolite differences. Analysis of differential metabolites identified 112 significantly upregulated (fold change ≥ 2.0, *q* < 0.05) and 8 downregulated (*p* < 0.05) metabolic features in 7Li ion beam radiation samples Long 158 compared to controls (Figure 5A). KEGG pathway enrichment analysis of these altered metabolites showed predominant involvement in 4-Hydroxy-6-methyl-2-pyrone, N-epsilon-Acetyl-L-lysine, 1-Hydroxy-2-naphthoic acid, Zymosterol, m-cresol, urocanic acid, 2,6-Diaminopimelic acid, and Scopoletin (Figure 5B). Profiling of Long 158 versus Long 5 revealed notable alterations in amino acids, sugars, sugar alcohols, organic acids encompassing pathways for carbohydrate metabolism, amino acids biosynthesis, TCA cycle, and niacin/nicotinamide metabolism (Figure 5C). Comparative analysis suggested an absence of mutual inhibition between the pathways in both groups. Their concerted expression profiles likely play complementary roles in pivotal metabolic routes or fundamental cellular functions.

### 3.6. Correlation Analysis of Proteomic and Metabolome

**Figure 6:**
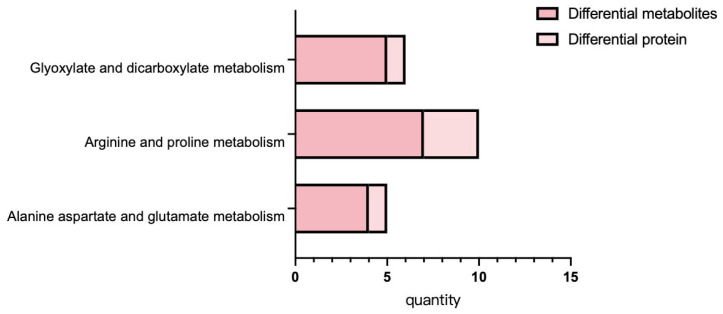
Metabolic pathway map of differential metabolites. Note: In the figure, the orange area is glucose metabolism, the blue area is amino acid metabolism, the green area is energy metabolism, the purple area is niacin and niacinamide metabolism, and the red border is marked with up-regulated compounds.

Integrated proteomic and metabolome data were displayed in Figure 6. These results indicate that differentially expressed proteins and metabolites are primarily involved in the following metabolic pathways: arginine and proline metabolism, alanine, aspartate and glutamate metabolism, and glyoxylate and dicarboxylate metabolism. Conjoint KEGG enrichment analysis revealed that there was significant co-enrichment in the photosynthesis and phenylpropanoid biosynthesis pathways across the proteomic and metabolomic datasets. Additionally, a subset of pathways showed interconnections, such as carbon fixation in photosynthetic organisms and photosynthesis, both of which are associated with energy metabolism.

Metabolite function is regulated by enzymatic activity, and enzymes are inherently proteinaceous. Therefore, we conducted further analysis based on shared pathways involving both divergent proteins and metabolite profiles, including amino acid metabolism, carbohydrate metabolism, and energy metabolism, to provide a more integrated interpretation of proteomic and metabolomic data. Metabolic pathway mapping of arginine and proline metabolism revealed heightened activity of associated proteins and metabolites following 7Li ion beam radiation treatment. As shown in Table 1, the alanine, aspartate and glutamate metabolism pathway, pyruvate, fumarate, citrate and succinate were upregulated, whereas carbamoyl-phosphate synthase large subunit was downregulated. Carbohydrate metabolism analysis (Table 2) revealed upregulation of key components in the glyoxylate and dicarboxylate metabolism pathway, including citrate, isocitrate, succinate, glycolate, pyruvate, and catalase.

In summary, the omics analysis we performed found coordinated changes in metabolites and proteins in many biological processes. The accumulation of key metabolites leads to changes in energy metabolism, which explains why plants become more stress-resistant after 7Li ion irradiation. This discovery links improvements in photosynthesis capacity with metabolic reprogramming, providing a new theoretical framework for pepper breeding.

### 3.7. Real-Time PCR Validation of DEGs in Capsicum annuum L.

**Figure 7:**
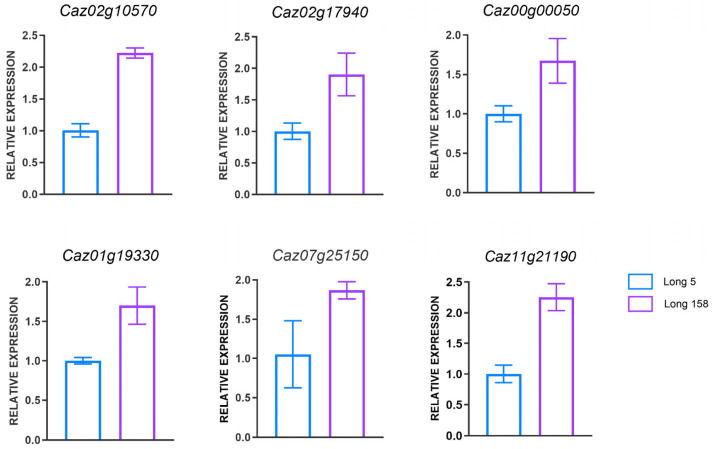
qPCR validation of pepper gene expression between Long5 and Long 158.

To validate our omics findings, we selected differentially expressed genes associated with phenylpropane and photosynthesis for experimental verification. Total RNA was extracted from the stems of seedling-stage Long 5 and Long 158 plants, reverse-transcribed into cDNA, and subjected to quantitative real-time PCR (qRT-PCR). Specific primers were designed for the photosynthesis-related genes *Caz02g10570*, *Caz02g17940*, *Caz00g00050*, *Caz01g19330*, *Caz07g25150*, and *Caz11g21190* (Supplementary Table S2). The relative expression levels determined by qRT-PCR confirmed the accuracy of the omics data. The results indicated that the expression levels of the six candidate genes in seedling stems were consistent with the proteomic data (Figure 7). That means validating the reliability of the proteomic sequencing results.

## 4. Discussion

As a mutagenic breeding technique that yields significant biological effects, 7Li ion beam radiation has been relatively underexplored in pepper breeding [20,21]. In this study, by analyzing growth status and stress-related physiological indicators following abiotic stress, along with integrated proteomic and non-targeted metabolomic profiling, we elucidate the regulatory mechanisms through which 7Li ion beam radiation enhances stress tolerance in pepper plants. We propose that the objective insights provided by proteomic and untargeted metabolomic analyses establish a foundation for understanding the mechanistic basis of stress-tolerance acquisition in 7Li ion beam radiation peppers.

### 4.1. Physiological Traits of Pepper Plants to 7Li Ion Beam Radiation

Physiologically, Long 158 plants subjected to 7Li ion beam radiation displayed visibly intensified stem pigmentation and significant increases in total chlorophyll, chlorophyll a, and chlorophyll b content. The chlorophyll a/b ratio serves as a key physiological trait sensitive to environmental changes. The elevated chlorophyll a/b ratio in radiated plants may indicate improved light capture capacity, potentially reflecting an adaptive photosynthetic optimization induced by 7Li ion beam radiation. Ling et al. [22] reported that 40 Gy ion beam irradiation stimulated chlorophyll accumulation in rice. Jia et al. [23] compared three distinct types of ionizing radiation and noted that while gamma ray and cosmic ray exposure resulted in slightly lower chlorophyll levels, 7Li ion beam radiation treatment led to a marked increase—findings consistent with our observations. In contrast, other studies have indicated that ion beam radiation can suppress chlorophyll biosynthesis in plants, likely due to disruption of the porphyrin ring within chlorophyll molecules [24,25,26]. This difference likely arises from substantial variations in experimental parameters, such as radiation type and dose, plant species, and developmental stages across different studies.

Anthocyanins are the key plant secondary metabolites that protect against microbial pathogens, insect herbivory, abiotic stress, and UV radiation, thereby enhancing plant stress tolerance. Anthocyanins are flavonoid compounds, and their biosynthesis is regulated primarily by the phenylalanine metabolic pathway [27]. In this study, anthocyanin content was significantly higher in Long 158 than Long 5; however, subsequent proteomic and metabolomic analyses did not identify differentially expressed proteins or metabolites directly associated with anthocyanin biosynthesis. Future work could therefore explore other omics approaches, such as transcriptomics or epigenomics, to uncover key regulatory genes governing anthocyanin accumulation.

### 4.2. Differential Protein and Metabolite Analysis

In the pepper samples of Long 5 and Long 158 subjected to 7Li ion beam radiation, several proteins involved in photosynthesis and phenylpropanoid biosynthesis were upregulated. Photosynthesis, a central metabolic process in plants, serves as a critical hub connecting primary and secondary metabolism [27,28]. Phenylpropanoid compounds play a dual role in plant disease resistance: they function both as inducible antimicrobial agents and as signaling molecules [29]. Moreover, phenylpropanoid metabolism represents one of the most critical biochemical pathways activated in plants during biotic stress responses [29]. This suggests that 7Li ion beam radiation enhances photosynthetic efficiency, and the upregulated proteins in this optimized photosynthetic process may facilitate carbohydrate metabolism and the efficient transport of metabolites, thereby ensuring sustained energy supply to the plant. Several proteins involved in photosynthesis and phenylpropanoid biosynthesis were upregulated. Furthermore, phenylpropanoids, key secondary metabolites, showed marked differences between the two cultivars, likely reflecting an overall stimulation of secondary metabolism induced by 7Li ion beam radiation.

### 4.3. Analysis of Differentially Expressed Genes in Phenylpropanoid and Photosynthetic Pathways Following 7Li Ion Mutagenesis

Following 7Li ion radiation, key enzymes in the phenylpropanoid pathway, including cinnamyl alcohol dehydrogenase and peroxidase, were upregulated. 4-Coumarate-CoA ligase (4CL), a pivotal enzyme in phenylalanine metabolism and phenylpropanoid biosynthesis, catalyzes the formation of various substrates for lignin monomer synthesis [30]. The upregulated expression of Caz02g17940 in Long 158 promotes lignin biosynthesis, thereby enhancing stress resistance, a finding consistent with the results reported by Cheng Li et al. [31]. The upregulation of cinnamyl alcohol dehydrogenase in stems further suggests that mutations in phenylpropanoid biosynthesis genes influence stem development.

Altered expression of genes involved in plant photosynthetic signal transduction can modify photosynthetic characteristics, ultimately leading to changes in plant growth morphology. For instance, La Camera et al. [29] reported that in the rice multi-tiller dwarf mutant hfa-1, the most significantly enriched pathway for differentially expressed genes was photosynthesis, with these genes associated with the synthesis of protein complexes such as photosynthetic reaction centers and light-harvesting pigment complexes. Notably, PsaK, PsaN, and PsaH were significantly upregulated, a finding consistent with our results. These genes encode essential components of the Photosystem I (PSI) reaction center. Mant et al. [30,32] demonstrated that Arabidopsis PsaK loss-of-function mutants exhibit reduced light-use efficiency and impaired growth. Similarly, Naver et al. [31,33] showed that PsaH is critical for electron transfer within PSI and helps maintain the stability of the PSI complex. Moreover, loss of the PsaN subunit disrupts energy transfer from LHCII (light-harvesting complex II) to PSI, leading to impaired state transitions and a reduced capacity to acclimate to varying light conditions or balance excitation energy distribution between the two photosystems [34].

## 5. Conclusions

This study employed pepper cultivars Long 158 (treated with 7Li ion beam radiation) and Long 5 (untreated control) as experimental materials. Biological assays revealed that irradiated plants exhibited intensified stem and leaf pigmentation, indicating enhanced pigment accumulation. Transmission electron microscopy further demonstrated ultrastructural modifications in chloroplasts, including structural fibrosis. Integrated proteomic and metabolomic analyses identified substantial compositional differences between the two groups: 250 metabolites were quantified, with 120 being differentially abundant (112 upregulated, 8 downregulated), enriched in 9 metabolic pathways. Proteomic profiling detected 6082 proteins, including 355 differentially expressed proteins (139 upregulated, 216 downregulated), enriched in 4 KEGG pathways. Joint analysis revealed coordinated enrichment of differential metabolites and proteins in pathways related to amino acid and carbohydrate metabolism. Genes associated with photosynthesis and phenylpropanoid metabolism, *Caz02g10570*, *Caz02g17940*, *Caz00g00050*, *Caz01g19330*, *Caz07g25150*, and *Caz11g21190*, were significantly upregulated. This expression pattern is consistent with the observed phenotypes in Long 158, including dwarfism, elevated chlorophyll content, enhanced photosynthetic capacity, and phenylpropanoid pathway genes and increased lignin accumulation. These findings indicate that 7Li ion beam radiation enhances metabolic activity, photosynthetic capacity, and stress tolerance in pepper plants.

## Figures and Tables

**Table 1:** Alanine, aspartate and glutamate metabolism-related proteins and metabolites.

protein	**Accession**	**Name**	**Flod Change**	***p*-Value**
	A0A2G2YUX8	carbamoyl-phosphate synthase	−1.63	0.0001
metabolin	**Name**	**Mutation Weight Value**	**Flod Change**	***p*** **-Value**
	Citrate	1.23	1.81	0.046
	Fumarate	1.12	1.24	0.0247
	Succinate	1.09	1.48	0.0239
	Pyruvate	1.20	2.05	0.0130

**Table 2:** Carbohydrate metabolism-related proteins and metabolites.

protein	**Accession**	**Name**	**Flod Change**	***p*-Value**
	A0A2G2YAT8	catalase (CAT1; CAT2)	1.78	0.0001
metabolin	**Name**	**Mutation Weight Value**	**Flod Change**	***p*** **-Value**
	Citrate	1.23	1.81	0.046
	Isocitrate	1.36	1.60	0.003
	Succinate	1.09	1.48	0.0239
	Pyruvate	1.20	2.05	0.0138

## Data Availability

The original contributions presented in this study are included in the article/Supplementary Materials. Further inquiries can be directed to the corresponding authors.

## Supplementary Materials

The following supporting information can be downloaded at: https://www.mdpi.com/article/10.3390/genes16121486/s1, Table S1: The evaluation criteria for grading plant tolerance to high temperature, drought, low temperature, and salt stress. Table S2: Primer sequences of qRT-PCR. Table S3: Non-targeted metabolomics identified 120 major different metabolites.
